# BTKbase, Bruton Tyrosine Kinase Variant Database in X-Linked Agammaglobulinemia: Looking Back and Ahead

**DOI:** 10.1155/2023/5797541

**Published:** 2023-07-31

**Authors:** Gerard C. P. Schaafsma, Jouni Väliaho, Qing Wang, Anna Berglöf, Rula Zain, C. I. Edvard Smith, Mauno Vihinen

**Affiliations:** ^1^Protein Structure and Bioinformatics, Department of Experimental Medical Science, Lund University, BMC B13, 221 84 Lund, Sweden; ^2^Institute of Biomedical Technology, University of Tampere, Tampere, Finland; ^3^Department of Laboratory Medicine, Translational Research Center Karolinska (TRACK), Karolinska Institutet, Karolinska University Hospital, SE-141 86 Stockholm, Sweden; ^4^Centre for Rare Diseases, Department of Clinical Genetics, Karolinska University Hospital, Solna, SE-171 76 Stockholm, Sweden; ^5^Department of Infectious Diseases, Karolinska University Hospital, Huddinge, Stockholm, Sweden

## Abstract

BTKbase is an international database for disease-causing variants in Bruton tyrosine kinase (*BTK*) leading to X-linked agammaglobulinemia (XLA), a rare primary immunodeficiency of antibody production. BTKbase was established in 1994 as one of the first publicly available variation databases. The number of cases has more than doubled since the last update; it now contains information for 2310 DNA variants in 2291 individuals. 1025 of the DNA variants are unique. The human genome contains more than 500 protein kinases, among which BTK has the largest number of unique disease-causing variants. The current version of BTKbase has numerous novel features: the database has been reformatted, it has moved to LOVD database management system, it has been internally harmonized, etc. Systematics and standardization have been increased, including Variation Ontology annotations for variation types. There are some regions with lower than expected variation frequency and some hotspots for variations. BTKbase contains, in addition to variant descriptions at DNA, RNA and protein levels, also laboratory parameters and clinical features for many patients. BTKbase has served clinical and research communities in the diagnosis of XLA cases and provides general insight into effects of variations, especially in signalling pathways. Amino acid substitutions and their effects were investigated, predicted, and visualized at 3D level in the protein domains. BTKbase is freely available.

## 1. Background

Bruton tyrosine kinase (BTK) is a cytoplasmic enzyme essential for B cell maturation [1]. Variations in BTK can lead to a rare primary immunodeficiency called X-linked agammaglobulinemia (XLA, MIM# 300755), which is characterized by low B cell numbers and lack of immunoglobulins, leading mainly to bacterial infections in patients [2]. Antibody substitution therapy is an efficient treatment but requires lifetime management.

*BTK* gene [3, 4] (MIM# 300300) codes for a protein that contains in addition to the catalytic protein kinase domain, in addition to the C-terminal catalytic, the following order: the pleckstrin homology (PH) domain, Tec homology (TH) region, Src homology 3 (SH3), and SH2 domains. The PH domain is a versatile docking domain that has numerous binding partners [1]. The TH region contains two parts; in the N-terminus, there is a 27-residue-long zinc finger motif, and in the C-terminus, there are two proline-rich regions [5, 6]. The SH3 domain recognizes polyproline type II structures. The SH2 domain is a binding module specific for recognizing phosphorylated tyrosine residues.

BTK is predominantly expressed in B lymphocytes, apart from plasma cells, and in myeloid cells. BTK expression in the B cell lineage is developmentally regulated. With the exception of T lymphocytes, all other hematopoietic lineages have been shown to express BTK [7]. BTK is critical for B cell development, differentiation, and signalling, and its expression is assumed to be a prerequisite for B cell proliferation and survival. Variations causing loss of BTK activity lead to lack of circulating B lymphocytes and inability to generate immunoglobulins of all classes and therefore absent humoral immune responses. Tight regulation of BTK expression is essential for normal B cell function.

In addition to the B cells in healthy individuals, BTK is needed for the survival of tumor B cells in hematological malignancies, such as chronic lymphocytic leukemia (CLL, MIM# 151400). Therefore, BTK inhibitors provide efficient treatments for several leukemias and lymphomas [8–10]. However, cancers can obtain resistance to drugs due to modification(s) of the inhibitor binding site [11–14].

BTK variations at position 481, especially p.C481S, are common in hematological cancers [13–15]. XLA-causing variations have not been detected in this position that lines ATP-binding pocket. p.C481S is a common drug resistance variant in patients with B lymphoproliferative disorders treated with BTK inhibitors. Because this variant has kinase activity, it should not cause XLA [11]. A CRISPR-Cas-generated knock-in mouse strain has a phenotype identical to wild-type mice [16]. Inherited, kinase-inactive p.C481 substitutions by arginine, glycine, phenylalanine, tryptophan, and tyrosine cause resistance to BTK inhibitors [11] and are expected to cause XLA and have been predicted to be disease-causing [17].

BTK is mainly cytoplasmic but binds to the plasma membrane during B cell activation. Once localized to the plasma membrane via PH domain interaction, BTK is activated by phosphorylation of Y551 by spleen-associated tyrosine kinase (SYK) or LYN proto-oncogene, Src family tyrosine kinase (LYN). Then, BTK phosphorylates and activates phospholipase C gamma 2 (PLCG2) [18], leading to Ca^2+^ mobilization and activation of key pathways, including the protein kinase C pathway with its effector, transcription factor nuclear factor kappa B subunit 1 (NFKB1), and mitogen-activated protein kinase (MAPK). BTK undergoes autophosphorylation at Y223. Inactivation of BTK signalling is controlled by dual serine/threonine phosphorylations in the PH and kinase domains, which attract 14-3-3 proteins targeting phosphorylated BTK for degradation [19].

BTKbase was originally established in 1994 [20], among the very first locus-specific variation databases (LSDBs) and the first one for primary immunodeficiencies (PIDs). Eight versions have been published during the years, all including novel features and improvements in addition to new variants. BTKbase led to the launch of related databases for several primary immunodeficiences (PIDs), including CD40 ligand [21], cytochrome b-245 beta chain (CYBB) [22], IL2RG [23], WAS [24], and over 100 PID databases [25]. It was also a model for domain-specific databases for Src homology 2 (SH2base) [26] and protein kinase domain (KinMutBase) variants [27, 28].

**Figure 1:**
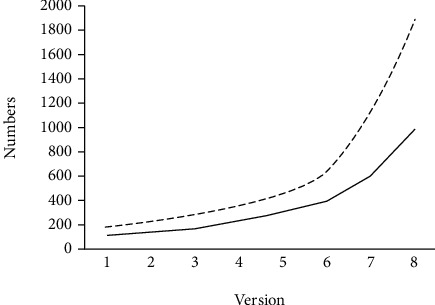
Growth of BTKbase, new cases per version (solid line), and a total number of cases (dashed line). This is the ninth release. The releases are related to published reports (see the text). Variants have always been made immediately available, apart from the confidential cases.

The database model, contents, and systematics were established during the first versions [20, 29, 30]. At that time, we also built the international network that supported the registry. Analysis of CpG dinucleotides was introduced to BTKbase version 4 [31] since they were found to be enriched among variants [32]. The following version [33] contained a number of new features including a submission tool which was introduced as part of the database management system MUTbase [34]. Putative structural implications of amino acid substitutions were introduced in the next version [35]. Then, BTKbase update was published together with other PID variation databases [36]. In year 2006, we added an extensive analysis of clinical and laboratory parameters relevant for XLA [37]. We have also published an extensive analysis of variations in the database [38]. BTKbase has grown more than 12-fold, from 188 variants in 1995 to the current number of 2310 (Figure 1). The ratio of unique variations has dropped somewhat from 65% in the first version to 44% in the current version. This ratio is considered very high, and it indicates a large proportion of *de novo* variations in the X-chromosomal disease. The database has proven most useful; it has helped in clinical diagnosis and contributed to research in many ways. BTKbase has been electronically distributed from the very beginning. Further, we have included extensive protein structural analysis and interpretation based on computational models, predictions, and experimental structures.

Here, we report a completely updated and renewed BTKbase. The extended data set facilitates improved clinical diagnosis, detailed analyses of the mechanisms of variations, and their effects and provides information about the involvement of the domains in BTK function and signal transduction in general.

## 2. Data Collection and Implementation of BTKbase

Novel cases are either from literature or obtained by direct submissions. Most of the variants are from literature. The genomic, mRNA, and protein reference sequences are from Locus Reference Genomic (LRG_128) [39]. The reported variants had to match the reference sequence; otherwise, they were omitted. For that reason, some variants are missing from certain publications. All the cases in BTKbase were manually curated in addition to some automatic checks.

The home for BTKbase (and the other IDbases) has moved to Lund University where it continues to be freely available at http://structure.bmc.lu.se/idbase/BTKbase. The variant data have been transferred to the LOVD database management system [40], available at https://databases.lovd.nl/shared/genes/BTK. During this process, numerous corrections and consistency checks were made. The database follows the published guidelines for LSDB establishment [41] and curation [42] and recommended standards and systematics including Human Genome Variation Society (HGVS) nomenclature [43], LRG reference sequences [39], which were agreed together with the LRG development team, and Variation Ontology (VariO) variation type annotations [44] that were generated by the automatic VariOtator tool [45]. The original IDRefSeq reference sequences [25] were replaced by those in LRG, and all variants were mapped on DNA, RNA, and protein levels, when relevant. We followed the HGVS variation nomenclature [43] for naming, obtained with Mutalyzer [46] or VariantValidator [47] tools, unless the experimental data differs from the automatically generated annotations. Some exceptions were made when the HGVS nomenclature did not allow exact description, especially for unsense variants [48], some DNA deletions, and protein truncations [49]. Unsense variants are a new category; these variants look synonymous but affect protein or protein expression due to aberrant splicing, modification of exonic splicing regulation site, or alterations to regulatory miRNA binding sites [48, 49]. Depending on exon, even more than 50% of variants annotated as synonymous may be unsense variants, and thereby, e.g., many evolutionary calculations need to be reconsidered [50]. The annotations of variants at the exonic and intronic splicing consensus regions were manually checked. This led to reclassifications of several cases, and thus the numbers of protein truncations were markedly reduced.

Variation Ontology [51] annotations for variation types were generated at the three molecular levels, when relevant. BTKbase follows also Human Variome Project (HVP) ethical guidelines and served as a model for some of the features of the guidelines [52].

During the transfer to LOVD, numerous consistency checks were performed, and corrections were made. BTKbase follows the HVP quality scheme and was preliminarily evaluated according to it [53]. BTKbase fulfills most of the quality criteria. Reported variations can be held confidential until published. The database contained some very old confidential cases. We tried to contact the submitters of these cases; some of them were made public, while others were deleted if the submitter could not be found or did not respond.

Variants causing premature stop codons were in the past annotated at protein level to cause protein truncations. In this version, truncations are annotated only if the premature stop appears in the last exon or within the last 50 bases in the penultimate exon (exon 18). Stop codons upstream of this position likely lead to nonsense-mediated decay, and thereby, the protein is not produced at all [49, 54]. These variants are now annotated at RNA level as “missing RNA” and at the protein level as “missing protein.” VariO annotations provide richer description than used before (see Table 1).

## 3. Variants

The updated BTKbase contains 2310 DNA variants, which represents a growth of 1199 variants (108%) since the last report [37]. This is the largest increase in the history of the BTKbase (Figure 1). The majority of the novel cases come from published literature; we also include a cohort of 108 patients from our cohort (Wang et al., in preparation). Some variants in the literature could not be included because unambiguous mapping to sequence position was not possible or there were errors in sequence information. In such cases, we tried to contact the authors but did not always get an answer. Sometimes these contacts have led to the publication of errata in journals and corrections to details in the database.

### 3.1. Variant Distribution and Characteristics

**Figure 2:**
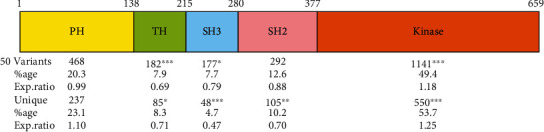
Variants in BTK domains. Numbers above the schema indicate the borders of domains and regions. The distribution of all variations and unique variations is indicated under the schema for the domain organization. The statistical significance for the enrichment of variations within the domains was calculated with chi square test; ^∗^*p* < 0.05, ^∗∗^*p* < 0.01, and ^∗∗∗^*p* < 0.001. The first number on the first text line indicates numbers of variant upstream of the coding region.

The XLA variants in BTK are distributed along the entire gene and protein (Figure 2); however, the distribution is not entirely even. The TH region, SH3, and SH2 domains have somewhat fewer variants than expected, whereas in the kinase domain, there is over abundance of variations in comparison to expectation.

Variants are annotated with VariO variation type classes in Table 1 in a systematic and unambiguous way. The numbers are given at DNA, RNA, and protein levels. To our knowledge, this is the first time this detailed and systematic description of LSDB contents is presented.

We used VariO terms as even some widely applied genetic terms are problematic [48, 55]. The variation type annotations were produced automatically by VariOtator [45] except for some splice site variants and other rare events that were manually annotated. Note that variants appear several times in Table 1 as the annotations are separate for DNA, RNA, and protein levels, and the same variant is described by several annotations. For example, RNA substitutions are further specified as transitions or transversions and are further classified as nonsense, missense, synonymous, or unsense substitutions or as splice site alterations [48]. The number of DNA variants is larger than the number of affected individuals, as some individuals have more than one variant in the *BTK* gene.

For the analysis of the variants presented here, we excluded from LOVD database VKGL classification records variants not related to XLA or not having any effect, those classified as benign or VUS (variant of unknown significance) and those of somatic origin. The presented data are exclusively for XLA.

The largest portion of the variants affects the kinase domain (49.4/53.7% for variants and unique variants, respectively) (Figure 2), which is also the largest (encompassing 42.8% of the BTK protein) and the most conserved domain. Substitutions are the most common alterations (72.1%) at DNA level, while missing RNA is the most abundant variant type on RNA (46.0%) and missing protein on protein level (47.1%) (Table 1). The numbers of annotated variants differ between the molecular levels. All the variants are annotated at DNA level, at RNA level 97.1%, and at protein level 93.8%. The cases of missing mRNA and/or protein annotations are e.g., in 5′noncoding region and the outcomes of the variants at RNA or protein level are not known.

Amino acid substitutions are very rare in any SH3 domain. Two amino acid substitutions, p.L216P (direct submission) and p.L222P [56], have been reported. The relevance of the former variant in XLA is not clear as the patient has totally six variants in the *BTK* gene.

Several of the arginine codons in BTK are well-known mutation hotspots due to the presence of CpG dinucleotides [32]. Only some of the 34 arginines in BTK frequently harbour variants, and even then, only certain substitutions are enriched. For detailed discussion, see [26, 31, 57].

18 individuals have an initiation codon substitution preventing protein production. These are now classified as protein missing, since no protein is produced. There are deletions, indels, and insertions on DNA 490/25/128, RNA 423/25/113, and protein 105/17/2, respectively. Protein deletions include 36 truncations. On the RNA level, they are divided into two categories: inframe and out-of-frame categories. The database contains inframe variations as follows: 132 deletions, 5 indels, and 3 insertions. The corresponding numbers for out-of-frame variants are 334, 20, and 110. These changes lead to either missing protein, sequence retaining, or amphigoric (mRNA reading frame destroying) alterations at protein level. The latter type originates from DNA alterations that are not divisible by three and therefore changing the RNA reading frame and most often causing premature chain termination. If the termination codon appears upstream of the 50th position from the end of the penultimate (18th exon), the transcript is likely detected by nonsense-mediated decay quality control mechanism and leads to missing protein [54].

There are 79 sequence retaining deletions, 17 indels, and 2 insertions. Indels contain both inserted and deleted sequence stretches. The differences in the numbers of variants at different levels are because the effects of variants may vary depending on the level. For example, a protein indel may originate from a nucleotide-level deletion affecting the reading frame. These numbers are smaller in comparison to previous BTKbase versions because they are now annotated with systematic VariO terms. On protein level, the largest protein variation category is missing protein with 1089 variants. In these cases, the protein is not translated due to premature stop codon, e.g., due to splicing error or RNA nonsense variation. The second largest group is amino acid substitution accounting for 943 instances (40.8%).

RNA variants can affect the coding region. The database contains altogether 1063 missense, 380 nonsense, and 6 unsense variants. RNA molecules with nonsense variants locating two or more exons away from the C-terminus are considered to be destroyed by NMD. Consequently, the protein production is prevented. There are 35 truncating protein variants that escape mRNA NMD due to being close to the end of the sequence. There may be additional NMD escape cases, likely among patients with milder forms of XLA.

Tables 2 and 3, respectively, show the distributions of nucleotide and amino acid substitutions. When considering the amino acid substitutions, only 150 of the 380 replacements are possible due to single nucleotide alterations. Changes of two or three bases within a single codon are extremely rare.

Substitutions to pyrimidines are somewhat more common than those to purines, 52.3% vs. 47.7%. Transitions (61.9%) are more common than transversion, similar to the previous versions of the database. The CpG dinucleotide is the single most common varied sequence [32] and was observed already in early versions of BTKbase [31]. G to A and C to T transitions account altogether for 47.7% of all the substitutions. As CpGs appear in four out of the six codons for arginine, replacements of this long and charged residue are the most frequent, accounting for 34.2% of all the amino acid substitutions in BTKbase.

Leucine (9.9%) is the second most commonly replaced residue. Among the substituting residues, proline (11.8%) is the most common, followed by tryptophan (10.1%), histidine (8.2%), glutamine (7.8%), and cysteine (7.7%). Due to its ring structure involving the amino acid backbone, proline is a special amino acid, and therefore, it is not compatible with the native structure in many positions. Substitutions by tryptophan, the bulkiest and largest amino acid, are often harmful because of collisions with other residues and consequent structural alterations.

During the conversion of the database to LOVD, splice site annotations were remade. Some of the RNA annotations are based on experimental studies; the remaining ones are predictions. Variations at consensus splice sites practically always impair the splice sites. Several variations recorded previously as amino acid substitutions or synonymous variants in the exons at splice sites are now annotated as splicing variants or unsense variants. Exon skipping is possible only in the case of exons 3, 4, 6, and 9 without changing the reading frame. Intron retention for all the introns in *BTK* leads to frameshift alterations, premature termination codons, and likely degradation by RNA quality control mechanisms.

### 3.2. Structural Consequences of Variations

**Figure 3:**
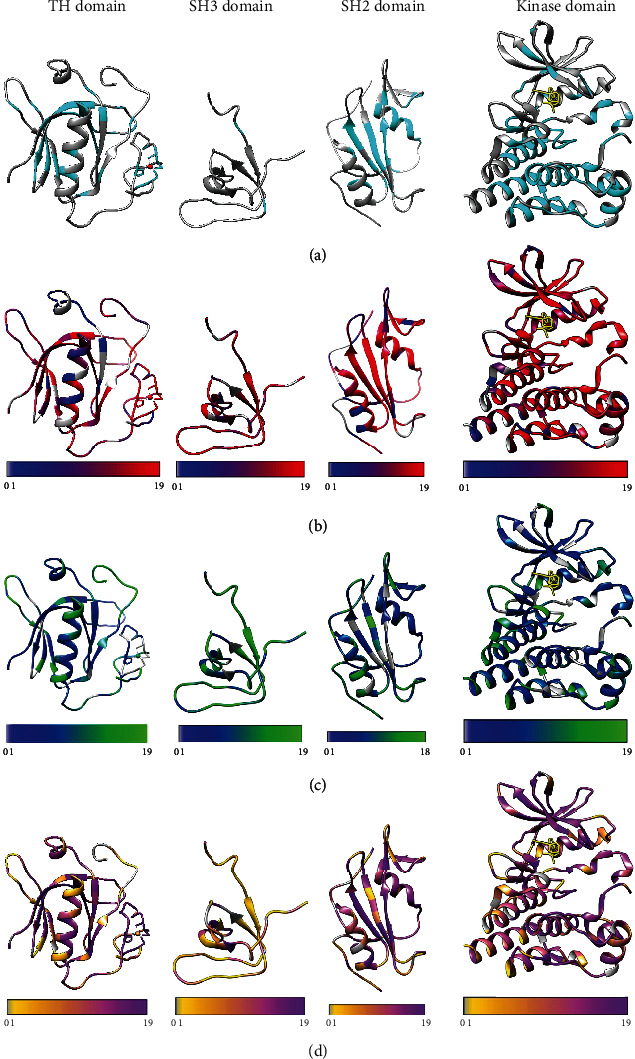
Mapping of BTK variants to domain structures: (a) positions of XLA-causing variants in BTK domains, (b) numbers of predicted pathogenic variations obtained with PON-P2, and (c) numbers of predicted mild/moderate and (d) predicted severe XLA-causing variants according to PON-PS in BTK domain structures. (b–d) All 19 variations in each position were predicted, and there is a scale describing the colours. The domains are, from left to right, PH, SH3, SH2, and kinase domain. The PDB structures used were 1btk for PH [63], 1awx for SH3 [64], 2ge9 for SH2 [65], and 5p9j for the kinase domain [66]. Zn^2+^ ion in TH region in the PH domain structure is shown as a sphere and inhibitor ibrutinib in the kinase domain with yellow stick presentation.

BTK consists of five domains (Figure 2); the three-dimensional structure is known for four domains and the N-terminal half of the TH region. The TH region is specific for this kinase family [5, 6]. The C-terminal half contains proline-rich segments which can bind intramolecularly to the SH3 domain [58, 59]. The C-terminal end of the TH region contains two polyproline type II segments and is apparently very flexible. Therefore, we predicted the structure of BTK with AlphaFold2 [60], which has shown good performance also on intrinsically disordered proteins and regions [61]. In addition, we used Database of Disordered Protein Predictions (D^2^P^2^) [62]. Based on these predictions, the C-terminal part of the TH region highly likely does not have a stable ordered structure, which explains why an experimental structure has not been obtained for any complete Tec family TH regions despite numerous trials. This may well be the reason for the lack of complete BTK structure despite several attempts. The TH region, similar to other disordered regions, can have several conformations that facilitate binding to numerous partners. The N-terminal end of the TH region has an ordered Zn finger structure, where several XLA-causing variants affect the Zn^2+^ binding residues (Figure 3(a)).

The structural analyses in Figure 3 were based on experimentally defined structures for the PH, SH3, SH2, and kinase domains, and PDB codes 1btk [63], 1awx [64], 2ge9 [65], and 5p9j [66], respectively. All the structures were visualized with the UCSF Chimera [67].

The distribution of the known XLA-related amino acid substitutions in the domains is shown in Figure 3(a). Many of the sites are involved in different functions, such as ligand binding and catalysis, as well as posttranslational modifications. Changes to buried sites are more frequently disease-related than exposed sites and organized secondary structural elements more often than loop regions in BTK (see also (Wang et al. in preparation)). The actual effects depend on the type of variation as well as the context and function of the wild-type residue.

We have predicted the pathogenicity of all the single nucleotide change-caused amino acid substitutions (SNAVs) in the BTK kinase domain [17]. 67% of the substitutions were predicted to be harmful. This is a relatively high number; however, it is considered to be reliable, because the kinase domain has numerous functions and thereby has restrictions and requirements for amino acids, e.g., in ligand and ATP binding, regulatory phosphorylation and concomitant major structural alteration, and in interactions with other parts of the molecule and partners.

When we investigated all the possible single amino acid substitutions in the BTK kinase domain with highly reliable PON-P2 predictor [68], the number of pathogenic variants was found to be 73% (Schaafsma and [69]). Figure 3(b) shows the distribution of all predicted harmful variants in the BTK domains. When these results are compared to the known disease-causing variants (Figure 3(a)), it becomes apparent that many of the known variants affect hotspots of important residues where many or practically all substitutions would be harmful. Despite the large number of variants compiled to the BTKbase, the known XLA cases cover just a small fraction of the possible variation landscape; therefore, predictions are essential.

The disease phenotype for BTK variants can vary from severe (classical) XLA to moderate and mild forms. Sometimes both severe and mild/moderate variations can emerge in the same positions, depending on the substitution type. There is also phenotypic heterogeneity; the severity of the diseases caused by the same variant may differ in different individuals.

We predicted the severity of all the possible BTK single amino acid substitutions, i.e., 19 substitutions in each position. The results are for mild and moderately severe variants in Figures 3(c) and 3(d) for predicted severe variants. The predicted benign variants are shown for each domain in Supplementary Figure 1. These results were obtained with PON-PS predictor [70]. Note that the colours in Figures 3(b)–3(d) and Supplementary Figure 1 display a range according to the number of predicted cases per position. The keys for the colours are shown in each panel.

## 4. Biological Relevance

BTK variations do not display strong genotype-phenotype correlations; however, some correlations exist, as previously discussed [37, 57, 71–73].

BTK is crucial for B cell development; thus, the large number of different variants has highlighted the function and processes of BTK. Genes for protein kinases form one of the largest gene families in human. Numerous protein kinases are known to be involved in diseases. As BTK contains the largest number of different variants among human kinases, this information has been instrumental for understanding variations in almost all cellular signalling processes. BTK has contributed significantly to cellular signalling studies as it is a central regulator in several pathways and there are disease-causing variants in all the protein domains and regions, thus indicating several ways how the protein function can be impaired.

## 5. Clinical Relevance

The hallmark XLA characteristics are rudimentary B cell areas in lymphoid organs, profoundly reduced B cell numbers in all locations, very low Ig levels of all classes, and lack of specific humoral immune responses. On top of that, other signs and symptoms occur. Previous update extensively discussed the clinical relevance and features [37]. Clinical parameters in the new articles confirm the previous observations. The most common new information is for immunoglobulin and B cell numbers.

The majority of the patients have markedly reduced levels of B cells and immunoglobulins. Our analysis [37] showed reduced numbers of CD19^+^ and CD20^+^ cells as well as immunoglobulins IgA, IgG, and IgM in the patients. Individuals show heterogeneity; 11% of the patients had levels of the three immunoglobulins within near normal ranges.

The severity of XLA varies between patients and even among patients having the same genotype. There are examples that homozygous twin brothers can have different phenotypes. XLA severity is usually divided into three categories: severe or classical, moderately severe, and mild form. The borders between the types are somewhat fuzzy. This is in line with our model for pathogenicity [69] that describes the continuum of pathogenicity.

We recently introduced the first generic predictor for variant severity in human proteins, PON-PS, that distinguishes between variants that cause benign, nonsevere, or severe phenotypes [70]. The method is based on machine learning and has been trained on known cases in many diseases. We applied the method to predict the severity of all possible amino acid substitutions in BTK. Figures 3(d) and 3(e) show the distributions of the phenotypic effects. The figure further facilitates comparison to sites of known XLA-causing variants as well as those predicted to be harmful, compared to Figures 3(a) and 3(b). The predictions are entirely independent and performed based on different types of data. Thus, correlations indicate the validity of these kinds of predictions.

## 6. Diagnostic Relevance

Information in BTKbase has been widely used to support diagnostic decisions. The included data are manually curated and are of high quality. Functional or other test results for the effects of the variants are not available for all cases. As XLA is an X-linked disease, harmful variants in boys are highly likely to be disease-causing due to full penetrance.

Information for the laboratory characteristics, such as immunoglobulin levels and B cell counts, indicates the ranges possible for patients [37]. Although clinical features are not provided for all cases, the large size of the database provides reliable distributions for the disease characteristics. Diagnosis can be based on the reduced numbers of CD19^+^ and CD20^+^ cells as well as on the levels of IgA, IgG, and IgM when below the normal range for age. However, this will not allow detection of all cases; a gene test is required for that.

## 7. Future Prospects

BTKbase was one of the first LSDBs, and it was also among the first ones available on the Internet. It has been widely used and frequently visited. The database is manually curated to guarantee the quality of data. Those steps that can be automated have been computerized to provide consistency.

BTKbase data have been used in numerous studies. There are a substantial number of citations to the various releases of the database. In addition, several reports have used the data but have not provided a citation or mentioned just the database URL. According to Google Scholar, there are collectively more than 800 citations to BTKbase, covering very wide spectrum of studies. The most common ones are reports of novel variants and case studies. Many papers are related to diagnosis and therapy, as well as epidemiology of XLA and PIDs in general. B cell biology is the topic in many studies, including those dealing with signalling pathways or autoimmunity. Protein structural studies form a substantial group of citations, including structure determinations, structural bases of diseases, molecular interactions, and structure-function and genotype-phenotype correlations. Since BTK is implicated in cancers, there are numerous investigations of inhibitors and drug resistance [13]. BTKbase data has been used in several bioinformatic applications including databases, method development, e.g., for variation interpretation and pathogenicity/tolerance prediction, and phylogenetic studies. Further, mRNA splicing has been the topic of many reports.

BTKbase was established already 29 years ago, and the need for it is just increasing. We are committed to continuing to maintain the database. The deluge of data from NGS studies is going to set new requirements for curation. The importance of variant interpretation will only increase. Because sequencing is so cheap, more genomes will be investigated and variants in them detected. However, the functional and other tests for the consequences of variants cannot be automated in the same way. Therefore, it is likely that we need to use computational approaches as well. Based on data in BTKbase and other sources, we have developed highly reliable methods for e.g. generic variant tolerance prediction, PON-P [74], PON-P2 [68], PON-All [75], and a BTK kinase domain specific tool, PON-BTK [17], based on data from BTKbase and other sources. Further, the data were involved in the development of a predictor, PON-PS, for phenotypic severity of the substitutions [70].

BTKbase will remain a trusted information source and help in clinical decision-making and diagnosis. A large portion of the database users searches information for the diagnosis of XLA cases. Our aim is to continue to provide open access to the database for the research and clinical communities.

## Figures and Tables

**Table 1:** Distribution of variation types at DNA, RNA, and protein level annotated with VariO terms.

	Number	% age
DNA		
DNA deletion (VariO:0141)	489	21.16
DNA indel (VariO:0143)	25	1.08
DNA insertion (VariO:0142)	128	5.54
DNA inversion	2	0.09
DNA substitution (VariO:0136), transition (VariO:0313), and purine transition (VariO:0315)	540	23.37
DNA substitution (VariO:0136), transition (VariO:0313), and pyrimidine transition (VariO:0314)	544	23.54
DNA substitution (VariO:0136), transversion (VariO:0316)	583	25.23
DNA substitution (total)	1667	72.13
Transition (total)	1084	46.91
Transversion (total)	583	25.23
RNA		
-	194	8.40
Inframe deletion (VariO:0320)	65	2.81
Inframe deletion (VariO:0320), RNA splicing change (VariO:0334)	24	1.04
Inframe indel (VariO:0030)	5	0.22
Inframe insertion (VariO:0332)	3	0.13
Intron gain (VariO:0364), missing RNA (VariO:0245)	1	0.04
Missing RNA (VariO:0245)	8	0.35
Out-of-frame deletion (VariO:0321)	17	0.74
Out-of-frame deletion (VariO:0321), missing RNA (VariO:0245)	294	12.73
Out-of-frame deletion (VariO:0321), RNA splicing change (VariO:0334), and missing RNA (VariO:0245)	14	0.61
Out-of-frame indel (VariO:0031)	1	0.04
Out-of-frame indel (VariO:0031), missing RNA (VariO:0245)	18	0.78
Out-of-frame insertion (VariO:0327)	648	28.05
Out-of-frame insertion (VariO:0327), missing RNA (VariO:0245)	97	4.20
Out-of-frame insertion (VariO:0327), RNA splicing change (VariO:0334), and missing RNA (VariO:0245)	7	0.30
RNA splicing change (VariO:0334)	17	0.74
RNA splicing change (VariO:0334), missing RNA (VariO:0245)	205	8.87
RNA substitution (VariO:0312), nonsense variation (VariO:0310), and missing RNA (VariO:0245)	361	15.63
RNA substitution (VariO:0312), transition (VariO:0313), purine transition (VariO:0315), and missense variation (VariO:0308)	309	13.38
RNA substitution (VariO:0312), transition (VariO:0313), and pyrimidine transition (VariO:0314)	3	0.13
RNA substitution (VariO:0312), transition (VariO:0313), pyrimidine transition (VariO:0314), and missense variation (VariO:0308)	317	13.72
RNA substitution (VariO:0312), transversion (VariO:0316), initiation codon change (VariO:0317), and missing RNA (VariO:0245)	18	0.78
RNA substitution (VariO:0312), transversion (VariO:0316), and missense variation (VariO:0308)	311	13.46
RNA substitution (VariO:0312), transversion (VariO:0316), and nonsense variation (VariO:0310)	13	0.56
Unsense variation (VariO:0514)	5	0.22
In-frame deletion (total)	84	3.64
Out-of-frame deletion (total)	325	14.07
Out-of-frame indel (total)	19	0.82
Out-of-frame insertion (total)	107	4.63
RNA splicing change (total)	62	2.68
RNA substitution (total)	1334	57.75
Missing RNA (total)	1333	57.71
Transition	628	27.19
Transversion		0.00
Nonsense (total)	374	16.19
Protein		
-	183	5.54
Amino acid substitution (VariO:0021)	935	0.09
Amphigoric amino acid indel (VariO:0023)	1	23.38
Missing protein (VariO:0240)	1058	23.55
Protein truncation (VariO:0015)	17	25.24
Protein truncation (VariO:0015), amphigoric amino acid indel (VariO:0023)	18	72.16
Sequence retaining amino acid deletion (VariO:0016)	79	46.93
Sequence retaining amino acid indel (VariO:0029)	19	25.24
Sequence retaining amino acid insertion (VariO:0020)	2	0.04
Protein truncation (total)	35	8.40

**Table 2:** Numbers and percentages of nucleotide substitutions.

Original	Variant				
	A	C	G	T	Total
A	—	48	128	58	234
C	77	—	44	382	503
G	413	84	—	138	635
T	56	163	77	—	296
Total	546	295	249	578	1668

	A	C	G	T	%age
A	—	2.88	7.67	3.48	14.03
C	4.62	—	2.64	22.90	30.16
G	24.76	5.04	—	8.27	38.07
T	3.36	9.77	4.62	—	17.75
%age	32.73	17.69	14.93	34.65	—

**Table 3:** Distribution and percentages of amino acid substitutions.

Original	Variant																				Total	% age
	A	C	D	E	F	G	H	I	K	L	M	N	P	Q	R	S	T	V	W	Y		
A			11	2									6				2	8			29	3.08
C					7	7									8	2			2	24	50	5.31
D				4		2	2					3						9		1	21	2.23
E			6			5			4					2							17	1.81
F		1								7						8		4		2	22	2.34
G	1	1	15	24											27	1		4	3		76	8.08
H			3							4		1	6	2	5					3	24	2.55
I					2						1	12			1	4	9				29	3.08
K				17				1				8			4						30	3.19
L					16			3					51	4	10	5		2	2		93	9.88
M								5	4	2					3		13	6			33	3.51
N									3											1	4	0.43
P	4									7					2	8	6				27	2.87
Q							3						2								5	0.53
R		41				25	60		5	9	2		14	65		11	2		88		322	34.22
S					6			2		3			20		3		1			6	41	4.36
T	3							4					12								19	2.02
V	4		7	1	10	2		1													25	2.66
W		11				3				2					6	2					24	2.55
Y		18	6				12					6				8					50	5.31
Total	12	72	48	48	41	44	77	16	16	34	3	30	111	73	69	49	33	33	95	37	941	
% age	1.28	7.65	5.10	5.10	4.36	4.68	8.18	1.70	1.70	3.61	0.32	3.19	11.80	7.76	7.33	5.21	3.51	3.51	10.10	3.93		

## Data Availability

The data are available in BTKbase at http://structure.bmc.lu.se/idbase/BTKbase/. The variation data are available at https://databases.lovd.nl/shared/genes/BTK.

## Supplementary Materials

Supplementary Materials Supplementary Figure 1 shows the numbers of predicted benign variants.
