# Supplementary material for: BTKbase, Bruton Tyrosine Kinase Variant Database in X-Linked Agammaglobulinemia: Looking Back and Ahead
Source: Hum Mutat. 2023 Jul 31;2023:5797541. doi: 10.1155/2023/5797541 (PMC11918983; doi:10.1155/2023/5797541)
Supplement: Supplementary Materials — Supplementary Figure 1 shows the numbers of predicted benign variants. [file 5797541.f1.docx]

**BTKbase, BRUTON TYROSINE KINASE VARIANT DATABASE IN X-LINKED AGAMMAGLOBULINEMIA – LOOKING BACK AND AHEAD**

Gerard C. P. Schaafsma^1^, Jouni Väliaho^2,*^, Qing Wang^3^, Anna Berglöf^3^, Rula Zain^3,4^, C. I. Edvard Smith^3,5^, Mauno Vihinen^1^

^1^Protein Structure and Bioinformatics, Department of Experimental Medical Science, Lund University, BMC B13, 221 84 Lund, Sweden

^2^Institute of Biomedical Technology, University of Tampere, Tampere, Finland

^3^Department of Laboratory Medicine, Clinical Research Center, Karolinska Institutet, Karolinska University Hospital Huddinge, 141 86 Stockholm, Sweden

^4^Centre for Rare Diseases, Department of Clinical Genetics, Karolinska University Hospital Solna, SE-171 76 Stockholm, Sweden

^5^Department of Infectious Diseases, Karolinska University Hospital, Huddinge, Stockholm,

Sweden

*Present address: Mylab Oy, Hatanpään valtatie 26, FIN-33100 Tampere, Finland


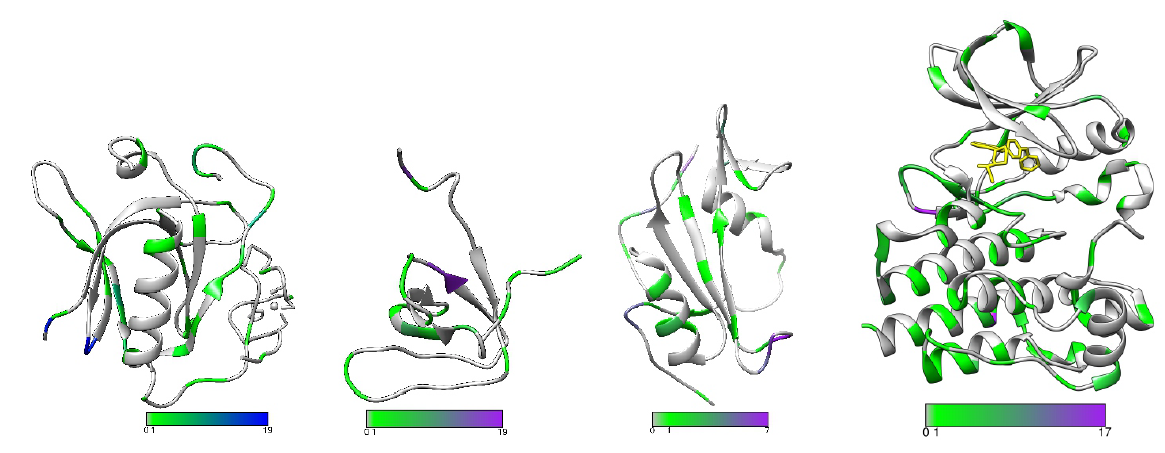


Supplementary Figure 1. Predicted benign variants in BTK domains. The predictions were obtained with PON-PS. The numbers of benign variants per position are color-coded and indicated by the scale for each domain. The variants shown in Figures 3C, 3D and in here sum up to 19.
